# The Eye-Heart Connection: Exploring the unique ocular features in tetralogy of Fallot

**DOI:** 10.22336/rjo.2025.91

**Published:** 2025

**Authors:** Airy Megha, Singh Kumar Basant, Singh Kumar Vinod, Kumar Santosh, Srivastava Anamika, Rawat Ayusha

**Affiliations:** 1 Moti Lal Nehru Medical College Campus, Prayagraj, Uttar Pradesh, India

**Keywords:** congenital heart disease, Tetralogy of Fallot, proliferative vitreoretinopathy, polymethyl methacrylate, intraocular pressure, CHD = congenital heart disease, TOF = Tetralogy of Fallot, PVR = proliferative vitreoretinopathy, PMMA = polymethyl methacrylate, IOP = intraocular pressure, RVOT = right ventricular outflow obstruction, IVTA = intravitreal triamcinolone acetate

## Abstract

This is a case report of an 18-year-old male who presented with a 7-year history of diminished vision in the right eye. Ophthalmological examination revealed light perception with accurate projection of rays in both eyes, broken posterior synechiae, and a membranous calcified cataract in the right eye. The left eye showed grade 2+ aqueous cells and proliferative vitreoretinopathy (PVR). A general examination revealed cyanosis, hypertrophic osteoarthropathy, and growth retardation. Echocardiography confirmed Tetralogy of Fallot (TOF). Blood tests showed elevated haemoglobin and RBC count. The patient was planned for cataract surgery with PMMA (polymethyl methacrylate) lens implantation and intravitreal triamcinolone acetate injection.

## Introduction

Congenital heart disease (CHD) is one of the most common congenital disabilities. It can be divided into two main categories: cyanotic and non-cyanotic. One of the most common cyanotic congenital heart defects is Tetralogy of Fallot (TOF), which accounts for approximately 7-10% of all CHDs [**1**].

In TOF, the RVOT (right ventricular outflow obstruction) leads to increased pressure in the right ventricle, causing a right-to-left shunt. This shunt allows deoxygenated blood to bypass the lungs and enter the systemic circulation, leading to systemic hypoxia and cyanosis. As the condition progresses, hypoxia stimulates compensatory mechanisms, such as increased polycythaemia [**2**]. However, chronic hypoxia can also have detrimental effects on other organ systems, including the eyes. Ocular manifestations of TOF can include cataract, retinal ischemia, and other structural changes in the eye [**3**]. Given the association between chronic hypoxia and these eye issues, regular ophthalmological evaluation is essential for patients with CHDs [**4,5**].

The importance of routine ophthalmological examinations in patients with congenital heart defects cannot be overstated. Early detection of ocular signs can help uncover previously unrecognized cardiac abnormalities, as eye findings may provide critical clues to a patient’s cardiovascular condition [**6**].

## Case report

An 18-year-old male presented to our clinic with a history of progressively diminished vision in his right eye for the past 7 years. Upon ophthalmological examination, his visual acuity was limited to light perception in both eyes, with accurate projection of rays in all quadrants. Extraocular movements were complete in all gazes, and no apparent ocular misalignment was observed.

**Fig. 1:**
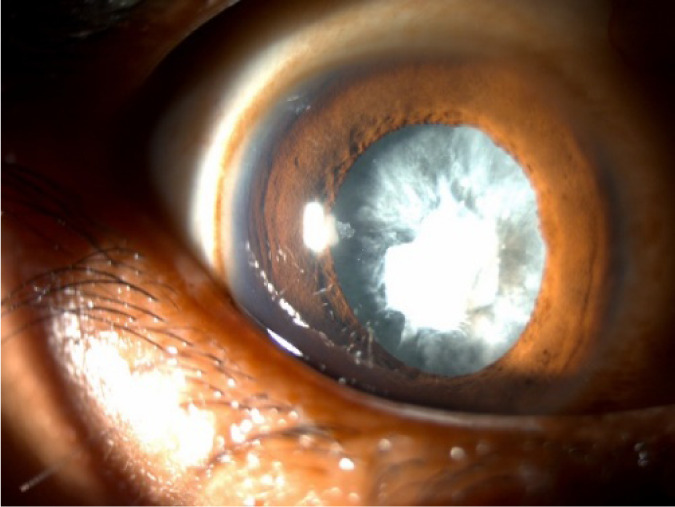
Right eye showing a membranous calcified cataract and broad-based synechiae at the 2 o’clock position

On slit-lamp examination, the right eye revealed broken posterior synechiae at the 2 o’clock position and a membranous cataract with anterior capsular opacification (**Fig. 1**). The left eye showed grade 2+ cells in the aqueous humour, suggesting mild anterior chamber inflammation. The anterior segment of both eyes otherwise appeared normal.

Fundus examination of the left eye showed proliferative vitreoretinopathy (PVR) (grade C, A1-12), which was also visible on B-scan ultrasound. Due to a cataract in the right eye, the fundus could not be directly examined, although the posterior segment appeared normal on B-scan. Intraocular pressure (IOP) was measured as normal in the right eye, but was too low to record in the left eye.

**Fig. 2:**
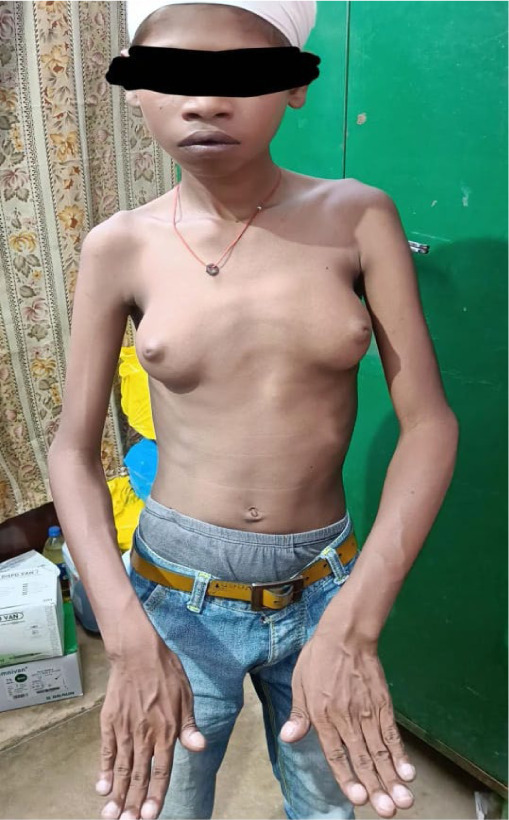
Gynecomastia and clubbing

**Fig. 3:**
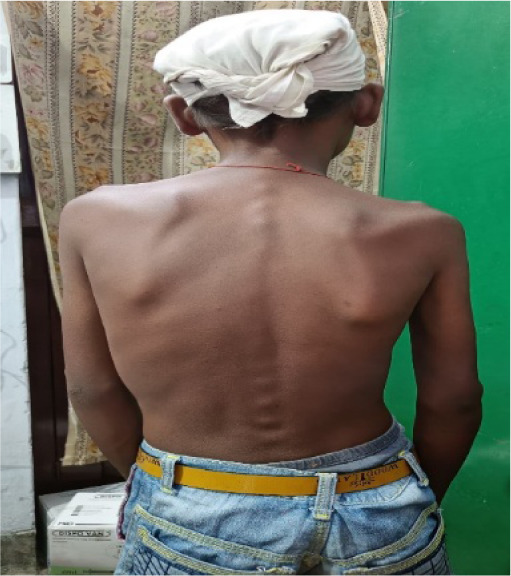
Back showing abnormal spinal curvature

General examination revealed cyanosis and hypertrophic osteoarthropathy in all four limbs. The patient’s weight was 27.7 kg, and his height was 140.5 cm, both of which were significantly below the third percentile for his age. He also had bilateral gynecomastia (**Fig. 2**). Skeletal evaluation showed thoracic scoliosis, loss of lumbar lordosis, and flat feet (**Fig. 3**).

**Fig. 4:**
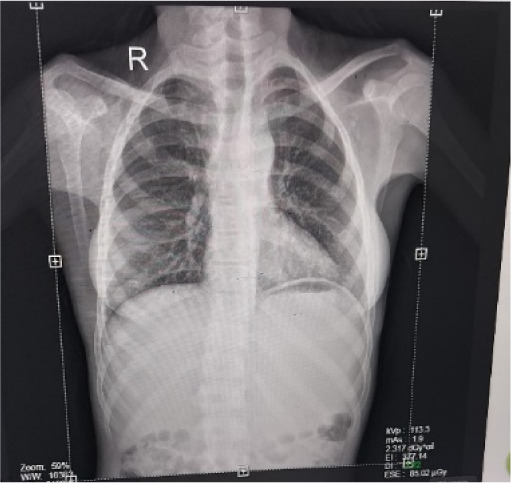
Chest X-ray of the patient showing increased concavity of the main pulmonary artery segment and syndesmosis of the right acromioclavicular segment

The patient was born through a normal full-term vaginal delivery with an uncomplicated prenatal course. He had two younger siblings, and there was no significant family history of congenital heart disease in the family. Chest X-ray showed syndesmosis of the right acromioclavicular joint (**Fig. 4**). Echocardiography confirmed the diagnosis of TOF.

Abdominal ultrasound revealed splenomegaly with portal vein dilation, though the liver parenchyma appeared normal. The kidneys were mildly echogenic but showed preserved corticomedullary differentiation. Routine blood tests indicated polycythaemia, with elevated haemoglobin (23.5 g/dL) and an increased red blood cell count (7.83 million/μL). His calcium level was low (5 mg/dL), which might indicate a secondary effect of chronic hypoxia.

Given the patient’s cataract and ocular inflammation, he was scheduled for small-incision cataract surgery, with implantation of a rigid PMMA lens and an intravitreal triamcinolone acetate (IVTA) injection to manage inflammation.

## Discussion

Patients with congenital heart disease, particularly those with cyanotic defects like TOF, are at an increased risk of developing ocular pathologies. These may include retinal vascular tortuosity, optic disc hypoplasia, congenital cataracts, strabismus, and retinal haemorrhages. The underlying cause of these eye problems is often chronic systemic hypoxia, which affects the retina and other ocular structures.

Several genetic syndromes like velocardiofacial syndrome (22q11.2 deletion syndrome), DiGeorge syndrome, and Down syndrome are frequently linked to both heart defects and eye problems, making early and comprehensive evaluation crucial [**7,8**]. Ocular findings can be an important clue in diagnosing these syndromes, and ophthalmologists should be vigilant in screening CHD patients for ocular complications.

## Conclusion

This case report underscores the importance of recognizing the interconnectedness of cardiac and ocular health in patients with congenital heart disease, particularly those with cyanotic defects like Tetralogy of Fallot. Ocular abnormalities in such patients can serve as an early indicator of cardiac dysfunction or hypoxia, making routine ophthalmological assessments an essential part of their care. With increasing survival rates due to modern cardiac interventions, it is vital to adopt a multidisciplinary approach to the long-term management of CHD patients, ensuring comprehensive care that addresses both their cardiac and ophthalmological needs.
